# Socioeconomic Disparities in Cardiovascular Health: A Cross-Sectional Analysis Unpacking the Sequential Mediation Roles of Protein Intake and Handgrip Strength

**DOI:** 10.3390/healthcare14131897

**Published:** 2026-06-30

**Authors:** Youlim Kim, Sun-Young Park

**Affiliations:** 1 College of Nursing, Kosin University, Pusan 49104, Republic of Korea; goshima0000@gmail.com; 2 College of Nursing, Kyungpook National University, Daegu 41566, Republic of Korea

**Keywords:** social determinants of health, social class, cardiovascular diseases, proteins, hand strength, mediation analysis

## Abstract

**Highlights:**

**What are the main findings?**

- Within the social determinants of health framework, this study suggests that protein intake and handgrip strength (HGS) sequentially mediate the relationship between socioeconomic status (SES) and 10-year atherosclerotic cardiovascular disease (ASCVD) risk using data from the 7th Korea National Health and Nutrition Examination Survey.
- Individuals with lower SES had lower protein intake and weaker HGS, which were associated with higher 10-year ASCVD risk.

**What are the implications of the main findings?**

- Our findings highlight the need for integrated public health interventions that address both dietary protein intake and muscle-strengthening strategies in socioeconomically vulnerable populations.
- Incorporating nutritional status and muscle strength into ASCVD risk assessment may support more equitable prevention policies and reduce health disparities.

**Abstract:**

**Objectives:** This study investigated the sequential mediating roles of protein intake and handgrip strength (HGS) in the association between socioeconomic status (SES) and 10-year atherosclerotic cardiovascular disease (ASCVD) risk within the social determinants of health (SDOH) framework. **Methods:** We conducted a complex survey analysis using data from 6281 adults without baseline ASCVD from the Korea National Health and Nutrition Examination Survey (2016–2018), accounting for stratification, clustering, and weighting. The 10-year ASCVD risk was estimated using the pooled cohort risk equations. SES was classified into three groups based on educational attainment and household income. Relative HGS was calculated as the sum of maximal grip strength in both hands divided by body mass index. Indirect effects were assessed using bootstrap-based confidence intervals (CIs). **Results:** Compared with the low-SES group, the high-SES group showed a lower 10-year ASCVD risk in both the total effect model (B = −1.630, 95% CI: −2.250 to −1.010) and the direct effect model (B = −1.550, 95% CI: −2.170 to −0.927). The total indirect effect was also significant in both the high-versus-low and middle-versus-low SES comparisons. Protein intake and relative HGS partially mediated the association between SES and 10-year ASCVD risk, and the sequential mediation pathway was also significant. **Conclusions:** Within the SDOH framework, these findings suggest that nutritional and muscle-strength-related pathways may link socioeconomic disadvantage to ASCVD risk and support integrated interventions targeting nutritional support and muscle-strength improvement for life-course prevention.

## 1. Introduction

Noncommunicable diseases, including cardiovascular disease, cancer, and chronic respiratory diseases, are the leading causes of death worldwide, including in South Korea [1,2]. In particular, cardiovascular disease accounts for approximately 17.9 million deaths annually worldwide [3]. According to statistics on the incidence of cardiocerebrovascular diseases in South Korea, there were 67.4 cases of myocardial infarction and 212.2 cases of stroke per 100,000 people in 2021; 16.0% of myocardial infarction cases and 19.3% of the stroke cases resulted in death within 1 year [4]. Because these diseases can be life-threatening if not diagnosed or treated early, are more costly to manage than many other conditions, and often lead to long-term disability due to sequelae, they impose a substantial burden on individuals and society.

The primary risk factors for ASCVD are smoking, unhealthy diet, physical inactivity, and harmful alcohol use. Although these behavioral risk factors are important, they do not occur in isolation; rather, they are shaped by broader social and structural conditions. From the perspective of the social determinants of health (SDOH), ASCVD risk is also shaped by upstream SDOH, including structural factors such as socioeconomic status (SES) [5]. SES reflects both economic and social position and includes indicators such as education, income, and occupation; it is generally positively associated with health status [6]. Studies [7,8,9] have shown that individuals with lower income or lower education attainment experience disproportionately higher rates of chronic disease-related mortality than those in higher-SES groups, underscoring the role of SES in shaping health outcomes and disease risk. More specifically, the association between socioeconomic circumstances across the life-course and health in later life has been explained by several hypotheses, including the accumulation model [10]. These models suggest that disparities in SES are a key mechanism underlying the development of chronic diseases, including ASCVD in adulthood. Therefore, it is important to examine the complex pathways through which socioeconomic background throughout the life course may shape modifiable health-related factors, such as dietary intake and muscle strength, which are subsequently associated with cardiovascular health outcomes.

From the perspective of the SDOH [11], SES functions as a structural determinant that shapes individuals’ access to health-related material and behavioral resources. Lower SES is associated with reduced access to nutritious foods, which may contribute to insufficient protein intake [12]. Higher income levels, for example, have been associated with increased protein and lipid intake, lower carbohydrate intake, and differences in the consumption of animal-based foods [13,14]. Inadequate protein intake, which is essential for muscle development and maintenance, may accelerate sarcopenia and reduce muscle strength [15,16]. Handgrip strength (HGS), a widely used indicator of overall muscle function, has been used to diagnose sarcopenia, assess frailty, and predict adverse health outcomes, such as falls, diabetes, osteoporosis, and metabolic syndrome [17]. Moreover, a longitudinal study of 142,861 individuals from 17 countries reported that lower HGS was associated with increased risks of ASCVD and mortality [18]. Collectively, protein intake and HGS may serve as intermediate factors linking structural socioeconomic inequalities to cardiovascular risk. Clarifying the proposed pathway from SES to protein intake, HGS, and cardiovascular risk may improve our understanding of nutrition- and muscle-strength-related mechanisms underlying socioeconomic disparities in cardiovascular health.

Accordingly, we conducted a secondary analysis using data from the 7th Korea National Health and Nutrition Examination Survey (KNHANES), which was conducted from 2016 to 2018 [19]. The aim of this study was to examine whether protein intake and HGS sequentially mediate the association between SES and 10-year ASCVD risk among adults aged 40–79 years without a history of ASCVD.

## 2. Materials and Methods

### 2.1. Study Design and Participants

This cross-sectional study was conducted as a secondary analysis of data from the 7th KNHANES (2016–2018). Of the 24,269 participants in the dataset, we included 6281 adults aged 40–79 years with complete data for all variables required to calculate the 10-year ASCVD risk score, including age, sex, systolic blood pressure, diabetes status, smoking status, serum total cholesterol, and high-density lipoprotein cholesterol, as well as other study variables such as HGS and protein intake. Individuals who self-reported a prior diagnosis of angina, stroke, or myocardial infarction were excluded. This study followed the Strengthening the Reporting of Observational Studies in Epidemiology (STROBE) reporting guidelines [20].

### 2.2. Data Collection

KNHANES is a legally mandated survey conducted by the Korea Disease Control and Prevention Agency under the National Health Promotion Act. Raw data from the 7th KNHANES (2016–2018) are publicly available. To minimize potential bias and ensure national representativeness, we applied sample weights and accounted for the complex survey design. To address potential information bias, trained interviewers collected data using standardized protocols and validated questionnaires.

In the KNHANES, self-report questionnaires were used to collect data on age, sex, household income, educational attainment, alcohol consumption, smoking status, and physical activity. In addition, the survey included measurements of systolic and diastolic blood pressure, serum total cholesterol, serum high-density lipoprotein cholesterol, fasting blood glucose, diabetes status, hypertension treatment, HGS, and protein intake.

### 2.3. Measurements

#### 2.3.1. SES

Based on household income and educational attainment, SES was classified into three groups using a predefined classification approach adapted from a previous study [21]. Household income was categorized into four groups based on quartiles: low, lower-middle, upper-middle, and high. Educational attainment was classified into three groups: elementary school graduates or lower, middle school and high school graduates, and college graduates or higher.

Participants were then classified into SES groups by combining educational attainment and income level [21]. The high-SES group included those who simultaneously met the criteria of college graduation or higher and the high-income quartile. In contrast, the low-SES group included those who simultaneously met the criteria of elementary school graduation or lower and the low-income quartile. Participants who did not meet either criterion were classified as the middle-SES group. According to this classification, the distribution of socioeconomic factors in the high-, middle-, and low-SES groups was 903 (15.6%), 5019 (79.9%), and 359 (4.5%), respectively.

#### 2.3.2. ASCVD Risk

The 10-year ASCVD risk was calculated using the ASCVD Risk Estimator, which was developed from the Pooled Cohort Risk Equations presented in the 2013 American College of Cardiology (ACC) and the American Heart Association (AHA) guidelines [20]. The equation includes age, sex, systolic blood pressure (including antihypertensive treatment status), total cholesterol, high-density lipoprotein cholesterol, smoking status, and diabetes status [22,23].

The 2013 ACC/AHA guidelines recommend moderate-intensity statin therapy for the primary prevention of ASCVD when the 10-year ASCVD risk is ≥7.5% [24]. Accordingly, we classified participants with a risk of ≥7.5% as high-risk and those with a risk of <7.5% as low risk.

#### 2.3.3. HGS

HGS was measured using a digital grip strength dynamometer (T.K.K 5401; Takei Scientific Instruments Co., Niigata, Japan). Participants were screened for eligibility through visual inspection for any deformities or injuries of the arms, hands, or thumbs. Additional questionnaire-based screening assessed the history of hand or wrist surgery within the previous 3 months, previous surgery for arthritis or carpal tunnel syndrome, ability to participate in the survey, and the presence or worsening of hand symptoms, such as pain, stiffness, or swelling, within the previous seven days.

For HGS measurement, the maximum value from three measurements was recorded. During measurement, participants stood upright with arms hanging naturally at hip level, ensuring that the elbows and wrists were not flexed. The sum of the maximum HGS values for both hands was taken as the absolute HGS, and the relative HGS was calculated by dividing the absolute HGS by body mass index (BMI) [25].

#### 2.3.4. Protein Intake

Protein intake was assessed using the 24-h recall method, in which participants reported all foods and beverages consumed during the previous day. The adequacy of protein intake was assessed based on the Estimated Average Requirement (EAR; 0.73 g/kg/day) and Recommended Nutrient Intake (RNI; 0.91 g/kg/day) per unit body weight, according to the Korean Dietary Reference Intakes [26].

To assess the proportion of individuals with inadequate protein intake, participants were categorized into two groups: those with intake below the EAR or RNI and those with intake at or above the EAR or RNI [27].

#### 2.3.5. Measurement of Variables Used to Calculate ASCVD Risk

In this study, the 10-year ASCVD risk was calculated using systolic blood pressure, diabetes status, current smoking status, serum total cholesterol, and high-density lipoprotein (HDL) cholesterol values. All physiological indicators were measured by trained medical personnel in mobile examination centers following standardized KNHANES testing procedures.

Systolic blood pressure was measured in a resting state according to standardized procedures, and values adjusted for measurement environment differences across survey periods were used. Serum total cholesterol and HDL cholesterol were analyzed at accredited clinical laboratories.

For HDL cholesterol, measurement traceability assessment and adjustment procedures were performed according to the U.S. Centers for Disease Control and Prevention Lipid Standardization Program. Diabetes status and current smoking status were collected through standardized health questionnaires [28].

### 2.4. Data Analysis

Data were analyzed using IBM SPSS Statistics (version 26.0; IBM Co., Armonk, NY, USA) and R statistical software (version 4.3.1; R Foundation for Statistical Computing, Vienna, Austria). To account for the complex sampling design of the KNHANES, stratification variables, primary sampling units, and integrated sampling weights were incorporated into all analyses.

Continuous variables were presented as means and standard deviations, and categorical variables were presented as frequencies and percentages. Differences between socioeconomic groups were examined using the Rao–Scott corrected chi-square test for categorical variables. Complex sample regression analyses were conducted in R using the survey package. A complex survey design object was specified using the KNHANES stratification variable, primary sampling unit, and integrated sampling weight. Survey-weighted linear regression models were then fitted to examine the associations among SES, protein intake, relative HGS, and 10-year ASCVD risk.

The pathways linking SES, protein intake, relative HGS, and 10-year ASCVD risk were examined using serial mediation analysis based on the product-of-coefficients approach. SES was treated as a categorical exposure variable, with the low-SES group used as the reference category. Two dummy variables were created to estimate the middle-versus-low and high-versus-low SES comparisons separately. Protein intake was specified as the first mediator, relative HGS as the second mediator, and 10-year ASCVD risk as the outcome.

Three survey-weighted regression models were estimated for the mediation analysis. Model 1 examined the association between SES and protein intake. Model 2 examined the associations of SES and protein intake with relative HGS. Model 3 examined the associations of SES, protein intake, and relative HGS with 10-year ASCVD risk. Based on these models, the total effect, direct effect, total indirect effect, and specific indirect effects were estimated separately for the middle-versus-low and high-versus-low SES comparisons. The specific indirect effects included the pathway through protein intake, the pathway through relative HGS, and the sequential pathway through protein intake and relative HGS.

Indirect effects were calculated using the product of the relevant path coefficients. To incorporate the complex survey design into the estimation of indirect effects, a replicate-weight survey design object was created, and replicate estimates were obtained using the withReplicates() function in the R 4.3.1 survey package. Bootstrap standard errors and 95% confidence intervals were calculated based on 1000 replicate estimates.

Age, sex, marital status, and aerobic physical activity were included as covariates in the multivariable models based on prior research [17]. In contrast, systolic blood pressure, lipid levels, diabetes status, and smoking status were not additionally adjusted because the 10-year ASCVD risk score is a composite index derived from these variables. Adjusting components of the outcome variable could introduce overadjustment and distort the interpretation of the proposed mediation pathway. Statistical significance was set at *p* < 0.05.

### 2.5. Ethical Considerations

KNHANES is conducted by the Korea Disease Control and Prevention Agency, and all data are collected after obtaining written informed consent from the participants. All data were anonymized to protect personal information. We used the data after completing an online data-use compliance form and adhered to ethical guidelines throughout the analysis. Furthermore, we obtained exemption approval from the Institutional Review Board of Kosin University (approval number: 1040549-250704-SB-0041-01).

## 3. Results

### 3.1. General and Health-Related Characteristics of Participants

The general and health-related characteristics of the participants are presented in Table 1. The mean age of the participants was 53.03 ± 0.17 years, with 2473 men (46.8%). Most participants were married (84.7%) and had completed high school (2153, 35.9%) or were college graduates or higher (2325, 40.9%). Household income was classified as low, lower-middle, upper-middle, and high, with 1424 (22.3%), 1567 (24.7%), 1590 (25.8%), and 1700 (27.2%) participants in each group, respectively. Health-related characteristics showed a mean height of 163.51 ± 0.13 cm and a mean weight of 63.64 ± 0.17 kg. Absolute and relative HGS values were 61.37 ± 0.30 kg and 2.60 ± 0.01 m^2^, respectively. The average daily protein intake was 71.51 ± 0.64 g/day, and the mean 10-year ASCVD risk was 5.42 ± 0.09%. Furthermore, 26.2% of all participants were classified into the high-risk group for ASCVD. The EAR for protein intake was 46.46 ± 0.12 g/day, and the RNI was 57.92 ± 0.16 g/day.

### 3.2. Correlation Between Variables

The results of the correlation analysis are presented in Table 2. The correlation coefficients were calculated using weighted data. SES showed a positive correlation with protein intake (r = 0.067, *p* < 0.001) and a negative correlation with 10-year ASCVD risk (r = −0.099, *p* < 0.001), whereas no significant correlation was observed with relative HGS (r = −0.014, *p* > 0.05). Protein intake was positively correlated with relative HGS (r = 0.047, *p* < 0.01) and negatively correlated with 10-year ASCVD risk (r = −0.070, *p* < 0.001). Relative HGS was also negatively correlated with 10-year ASCVD risk (r = −0.050, *p* < 0.001).

### 3.3. Simple and Sequential Mediating Effects of Protein Intake and Relative HGS on the Relationship Between SES and 10-Year ASCVD Risk

**Figure 1:**
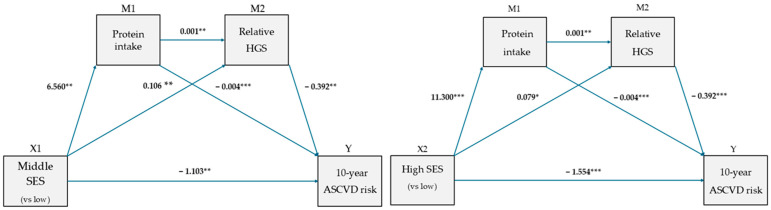
Sequential mediation model of the association between socioeconomic status, protein intake, relative handgrip strength, and 10-year ASCVD risk. * *p* < 0.05, ** *p* < 0.01, *** *p* < 0.001.

In accordance with the study hypothesis, we examined the simple and sequential mediating effects of protein intake and relative HGS on the relationship between SES and 10-year ASCVD risk (Table 3). Figure 1 graphically summarizes the sequential mediation pathways linking SES, protein intake, relative HGS, and 10-year ASCVD risk. Overall, the regression models supported the hypothesized pathway, showing that SES was associated with protein intake, protein intake was associated with relative HGS, and both protein intake and relative HGS were associated with 10-year ASCVD risk.

The left panel presents the comparison of middle SES versus low SES, and the right panel presents the comparison of high SES versus low SES. Path coefficients are shown on each arrow. SES, socioeconomic status; HGS, handgrip strength; ASCVD, atherosclerotic cardiovascular disease.

#### 3.3.1. Association Between SES and Protein Intake

Step 1 examined the association between SES and protein intake. The model explained 14.7% of the variance (R^2^ = 0.147, F = 9.163, *p* = 0.001). Compared with the low-SES group, protein intake was significantly higher in both the middle-SES (B = 6.560, *p* = 0.002) and high-SES (B = 11.300, *p* < 0.001) groups. Protein intake was negatively associated with age and female sex and positively associated with aerobic physical activity.

#### 3.3.2. Associations of SES and Protein Intake with Relative HGS

Step 2 evaluated the associations of SES and protein intake with relative HGS. The model explained 64.0% of the variance (R^2^ = 0.640, F = 6.343, *p* = 0.003). Compared with the low-SES group, both the middle-SES (B = 0.106, *p* = 0.001) and high-SES (B = 0.079, *p* = 0.047) groups were associated with higher relative HGS. Protein intake was also positively associated with relative HGS (B = 0.001, *p* = 0.008). Relative HGS was negatively associated with age, female sex, and unmarried status and positively associated with aerobic physical activity.

#### 3.3.3. Associations of SES, Protein Intake, and Relative HGS with 10-Year ASCVD Risk

Step 3 examined the associations of SES, protein intake, and relative HGS with 10-year ASCVD risk. The model explained 63.9% of the variance (R^2^ = 0.639, F = 17.762, *p* < 0.001). Compared with the low-SES group, the middle-SES (B = −1.103, *p* = 0.001) and high-SES (B = −1.554, *p* < 0.001) groups were associated with lower 10-year ASCVD risk.

Both protein intake (B = −0.004, *p* < 0.001) and relative HGS (B = −0.392, *p* < 0.001) were negatively associated with 10-year ASCVD risk. The 10-year ASCVD risk was positively associated with age and widowed or unmarried status and negatively associated with female sex and aerobic physical activity.

### 3.4. Mediation Analysis of the Association Between SES and 10-Year ASCVD Risk

The total effect of SES on 10-year ASCVD risk was statistically significant in both SES comparisons (Table 4). Comparing the high-SES group with the low-SES group, the total effect on 10-year ASCVD risk was B = −1.630 (95% CI: −2.250 to −1.010). Similarly, the middle-versus-low SES comparison yielded a total effect of B = −1.170 (95% CI: −1.770 to −0.575).

After adjusting for protein intake and relative HGS, the direct effects remained significant for both the high-SES (B = −1.550, 95% CI: −2.170 to −0.927) and middle-SES (B = −1.100, 95% CI: −1.700 to −0.507) groups, suggesting a pattern consistent with partial mediation.

The total indirect effects were statistically significant in both comparisons. For the high-versus-low SES comparison, the total indirect effect was B = −0.079 (95% CI: −0.130 to −0.038), whereas for the middle-versus-low SES comparison, it was B = −0.069 (95% CI: −0.115 to −0.036). The specific indirect effects through protein intake and through relative HGS were statistically significant in both SES comparisons.

Finally, the sequential indirect pathway linking SES to 10-year ASCVD risk via protein intake and relative HGS was statistically significant. The sequential indirect effect was B = −0.003 (95% CI: −0.006 to −0.001) for the high-versus-low SES comparison and B = −0.002 (95% CI: −0.004 to −0.0003) for the middle-versus-low SES comparison.

Sensitivity analyses with additional adjustment for smoking status and diabetes diagnosis produced similar results, with the direction and magnitude of the total, direct, and indirect effects remaining consistent, although the effect sizes were somewhat attenuated (Supplementary Table S1).

## 4. Discussion

Our findings support the SDOH framework, suggesting that socioeconomic inequalities may be linked to 10-year ASCVD risk through intermediate pathways involving protein intake and muscle strength. In our analysis, SES had a significant direct effect on 10-year ASCVD risk, with individuals in the middle- and high-SES groups exhibiting significantly lower 10-year ASCVD risk than those in the low-SES group. This finding is consistent with previous studies and supports the framework of structural health inequalities, which posits that lower SES is associated with adverse health outcomes [29].

Notably, a clear socioeconomic gradient in 10-year ASCVD risk was observed. The protective effect associated with socioeconomic resources was more pronounced in the high-SES group than in the middle-SES group, suggesting that greater socioeconomic resources may be associated with progressively lower risk as SES increases. This pattern indicates that socioeconomic vulnerability affects health not merely through a binary distinction between disadvantaged and advantaged groups, but across a continuous gradient. Consequently, public health policies should provide interventions tailored to varying risk levels across the entire socioeconomic spectrum.

Although this study employed a cross-sectional design, the findings are compatible with life-course perspectives, suggesting that socioeconomic disadvantage may be associated with adverse health outcomes over time [30]. This pattern can be interpreted in light of the weathering hypothesis, which proposes that prolonged exposure to socioeconomic adversity may lead to cumulative physiological strain over time [31]. However, because the present study did not directly measure longitudinal exposure, cumulative disadvantage, or physiological weathering, these concepts should be regarded as theoretical explanations rather than direct empirical findings. Within the SDOH framework, such disadvantages may be associated with nutritional status and muscle-strength-related markers. Specifically, the elevated 10-year ASCVD risk associated with low SES may reflect the cumulative impact of structural inequities, whereby socioeconomic disadvantage operates through intermediary determinants, including protein intake and muscle strength, which may be associated with greater cardiovascular vulnerability.

The indirect effect of SES on 10-year ASCVD risk mediated by protein intake was significant in both the high-versus-low and middle-versus-low SES comparisons, suggesting that socioeconomic disadvantage may be associated with reduced access to nutritionally adequate or high-quality diets, which may subsequently increase 10-year ASCVD risk. Limited food accessibility and insufficient protein intake are recognized risk factors among low-income populations [32]. Therefore, strategies to reduce disparities in protein intake across SES levels should be implemented through two key approaches: nutrition education and improved access to high-quality protein sources. Educational programs should focus on cost-effective and easy-to-prepare high-protein food options. Additionally, targeted support for vulnerable populations could be delivered through public food assistance programs, including community-based nutrition initiatives and health-center-based dietary support services.

Another noteworthy finding was that relative HGS, independent of protein intake, served as an additional mediating pathway linking SES to 10-year ASCVD risk. Although the bivariate correlation analysis showed no significant association between SES and relative HGS, the adjusted regression model indicated significant associations between SES and relative HGS in both the middle-versus-low and high-versus-low SES comparisons. This apparent discrepancy may be explained by the influence of covariates, particularly age and sex, which are strongly related to muscle strength. Therefore, the SES–HGS association observed in the regression model should be interpreted as an adjusted association within the proposed mediation framework, rather than as evidence of a strong unadjusted relationship. This pathway remained statistically significant in both the high versus low and middle versus low-SES comparisons, suggesting that muscle strength may function as an important protective factor against 10-year ASCVD risk across SES levels. Notably, the sequential pathway from protein intake to HGS was also significant, consistent with studies linking SES to HGS [33] and protein intake to muscle weakness and HGS [34,35]. These findings support the need for an integrated approach that emphasizes both nutritional and exercise interventions to improve muscle strength in strategies addressing health inequalities.

This study has some limitations. First, as a cross-sectional study, causal relationships between the variables cannot be definitively established. Second, protein intake data were collected using a 24-h dietary recall method, which may underestimate actual intake levels. Third, HGS was used as a measure of muscle strength, without assessing skeletal muscle mass or broader physical function indicators. Future studies should evaluate muscle strength using more precise and comprehensive measurement methods. Fourth, the outcome of this study was the estimated 10-year ASCVD risk rather than observed incident cardiovascular events. Therefore, the findings should be interpreted as associations with predicted cardiovascular risk, and future longitudinal studies are needed to determine whether these pathways are associated with actual ASCVD events. Fifth, 10-year ASCVD risk was estimated using the 2013 ACC/AHA Pooled Cohort Equations. Although these equations have been widely used in epidemiological studies, newer cardiovascular risk prediction algorithms such as PREVENT have recently been proposed. However, applying PREVENT would require redefining the analytic sample, incorporating additional predictors such as kidney function and lipid-lowering medication use, recoding medication-related variables, recalculating the primary outcome, and re-estimating the mediation models. Therefore, the use of PREVENT would substantially alter the study population and analytic framework. Future studies should examine whether similar mediation patterns are observed when newer cardiovascular risk prediction algorithms, including PREVENT, are applied to datasets specifically prepared for those models. Finally, the SES distribution was uneven, with a relatively small proportion of participants classified in the low-SES group. Because the low-SES group served as the reference category, this imbalance may have affected the stability of some estimates. Therefore, the SES contrast results should be interpreted with caution, and future studies should examine whether similar patterns are observed using alternative SES categorizations.

Despite these limitations, this study, based on the SDOH framework, identified a sequential mediating mechanism through which SES may be associated with 10-year ASCVD risk via protein intake and muscle strength. This finding is important because it provides empirical evidence for future policy interventions aimed at improving health equity. Overall, these findings highlight the need for targeted prevention strategies in clinical and community health services. Health professionals working in community settings are well-positioned to conduct early risk screening through regular assessments of dietary protein intake and muscle strength, and they play a pivotal role in designing and implementing community-based health promotion programs. Interventions that combine nutrition education—such as group workshops promoting cost-effective, high-protein diets—with supervised resistance training for older adults or at-risk adults may help reduce ASCVD risk in underserved populations.

## 5. Conclusions

Our analysis of the KNHANES data suggests that SES is directly and indirectly associated with the 10-year ASCVD risk. Protein intake and HGS served as sequential statistical mediators in the association between SES and 10-year ASCVD risk, highlighting the need for integrated health intervention strategies that combine nutritional support and muscle-strength improvement. To enhance health equity, public health interventions should consider prioritizing socioeconomically disadvantaged groups through integrated nutrition- and muscle-strength-based strategies, which may support ASCVD prevention efforts across the life course.

## Figures and Tables

**Table 1:** General and health-related characteristics of participants (N = 6281).

Variables	Mean ± SD	n (%)
Age (years)	53.03 ± 0.17	
Sex		
Men		2473 (46.8)
Women		3808 (53.2)
Education level		
Elementary school or less		1077 (13.0)
Middle school		726 (10.2)
High school		2153 (35.9)
College or above		2325 (40.9)
Household income		
Low		1424 (22.3)
Low middle		1567 (24.7)
Upper middle		1590 (25.8)
High		1700 (27.2)
Marital status		
Married		5274 (84.7)
Widowed		397 (5.0)
Divorced		364 (5.7)
Unmarried		246 (4.6)
Smoking		
Current smoker		977 (18.9)
Never/past smoker		5304 (81.1)
Aerobic physical activity		
Yes		2727 (45.4)
No		3554 (54.6)
Height (cm)	163.51 ± 0.13	
Weight (kg)	63.64 ± 0.17	
BMI (kg/m^2^)	23.71 ± 0.05	
Absolute HGS (kg)	61.37 ± 0.30	
Relative HGS (m^2^)	2.60 ± 0.01	
Protein intake (g/day)	71.51 ± 0.64	
EAR (g/day)	46.46 ± 0.12	
RNI (g/day)	57.92 ± 0.16	
Systolic BP (mmHg)	117.25 ± 0.28	
Diastolic BP (mmHg)	77.00 ± 0.17	
Total cholesterol (mg/dL)	200.85 ± 0.58	
LDL cholesterol (mg/dL)	119.48 ± 1.19	
HDL cholesterol (mg/dL)	51.26 ± 0.20	
Triglyceride (mg/dL)	144.16 ± 1.94	
10-year ASCVD risk	5.42 ± 0.09	
10-year ASCVD risk high-risk group		
≥7.5%		1931 (26.2%)
<7.5%		4350 (73.8%)
SES		
High		903 (15.6%)
Middle		5019 (79.9%)
Low		359 (4.5%)

Aerobic physical activity: At least 2 h and 30 min of moderate-intensity physical activity or 1 h and 15 min of vigorous-intensity physical activity per week, or moderate- and vigorous-intensity physical activity (1 min of vigorous-intensity activity followed by 2 min of moderate-intensity activity). SD, standard deviation; BMI, body mass index; HGS, handgrip strength; EAR, estimated average requirement; RNI, recommended nutrient intake; BP, blood pressure; LDL, low-density lipoprotein; HDL, high-density lipoprotein; ASCVD, atherosclerotic cardiovascular disease; SES, socioeconomic status.

**Table 2:** Correlation analysis among main variables.

Variable	SES	Protein Intake	Relative HGS	10-Year ASCVD Risk
SES	1			
Protein intake	0.067 ***	1		
Relative HGS	−0.014	0.047 **	1	
10-year ASCVD risk	−0.099 ***	−0.070 ***	−0.050 ***	1

Note. ** *p* < 0.01, *** *p* < 0.001. SES, socioeconomic status; HGS, handgrip strength; ASCVD, atherosclerotic cardiovascular disease.

**Table 3:** Survey-weighted regression models of the associations among SES, protein intake, relative HGS, and 10-year ASCVD risk.

Variables			Model 1. Protein Intake		Model 2. Relative HGS		Model 3. 10-Year ASCVD Risk	
			B (SE)	*p*	B (SE)	*p*	B (SE)	*p*
Independent	Middle SES (ref. Low)		6.560 (2.130)	0.002	0.106 (0.032)	0.001	−1.103 (0.331)	0.001
	High SES (ref. Low)		11.300 (2.650)	<0.001	0.079 (0.040)	0.047	−1.554 (0.342)	<0.001
Mediators	Protein intake		-	-	0.001 (0.001)	0.008	−0.004 (0.001)	<0.001
	Relative HGS		-	-		-	−0.392 (0.095)	<0.001
Covariates	Age		−0.510 (0.060)	<0.001	−0.017 (0.001)	<0.001	0.384 (0.008)	<0.001
	Female (ref. Male)		−23.920 (1.060)	<0.001	−1.250 (0.016)	<0.001	−4.693 (0.144)	<0.001
	Aerobic physical activity (ref. No)		4.700 (1.050)	<0.001	0.035 (0.015)	0.024	−0.392 (0.085)	<0.001
	Marital status (ref. Married)							
		Widowed	−1.970 (1.900)	0.299	−0.048 (0.030)	0.108	2.428 (0.410)	<0.001
		Divorced	−1.670 (2.340)	0.477	−0.014 (0.032)	0.659	0.036 (0.178)	0.840
		Unmarried	−5.370 (3.330)	0.108	−0.108 (0.050)	0.031	0.687 (0.152)	<0.001
Model fits			R^2^ = 0.147, F = 9.163 **		R^2^ = 0.640, F = 6.343 **		R^2^ = 0.639, F = 17.762 ***	

** *p* < 0.01, *** *p* < 0.001. SES, socioeconomic status; HGS, handgrip strength; ASCVD, atherosclerotic cardiovascular disease.

**Table 4:** Total, direct, and indirect effects of socioeconomic status on 10-year ASCVD risk mediated by protein intake and relative handgrip strength.

Effect	Path	Comparison	B	BootSE	95% CI
Total effect	X → Y	High vs. Low SES	−1.630	0.322	−2.250 to −1.010
		Middle vs. Low SES	−1.170	0.313	−1.770 to −0.575
Direct effect	X → Y	High vs. Low SES	−1.550	0.325	−2.170 to −0.927
		Middle vs. Low SES	−1.100	0.317	−1.700 to −0.507
Total indirect effect	All mediators	High vs. Low SES	−0.079	0.024	−0.130 to −0.038
		Middle vs. Low SES	−0.069	0.020	−0.115 to −0.036
Specific indirect effects	X → M1 → Y	High vs. Low SES	−0.045	0.017	−0.082 to −0.016
		Middle vs. Low SES	−0.026	0.011	−0.051 to −0.008
	X → M2 → Y	High vs. Low SES	−0.031	0.017	−0.068 to −0.003
		Middle vs. Low SES	−0.041	0.016	−0.078 to −0.012
	X → M1 → M2 → Y	High vs. Low SES	−0.003	0.001	−0.006 to −0.001
		Middle vs. Low SES	−0.002	0.001	−0.004 to −0.0003 ^†^

X = socioeconomic status (SES); M1 = protein intake; M2 = relative handgrip strength (HGS); Y = 10-year ASCVD risk. Indirect effects were estimated using bootstrap confidence intervals. BootSE, bootstrap standard error; SES, socioeconomic status; ASCVD, atherosclerotic cardiovascular disease. ^†^ Values are rounded to three decimal places, except for confidence interval limits close to zero, which are shown to four decimal places where needed.

## Data Availability

The data presented in this study are openly available in the Korea National Health and Nutrition Examination Survey (KNHANES) at https://knhanes.kdca.go.kr/knhanes/main.do (accessed on 28 April 2026), reference number [19].

## Supplementary Materials

The following supporting information can be downloaded at https://www.mdpi.com/article/10.3390/healthcare14131897/s1. Table S1: Sensitivity Analysis of Mediation Effects After Additional Adjustment for Smoking and Diabetes.

## Abbreviations

The following abbreviations are used in this manuscript:

ACC	American College of Cardiology
AHA	American Heart Association
ASCVD	Atherosclerotic cardiovascular disease
EAR	Estimated average requirement
HGS	Handgrip strength
KNHANES	Korea National Health and Nutrition Examination Survey
RNI	Recommended nutrient intake
SES	Socioeconomic status
