# Supplementary material for: Socioeconomic Disparities in Cardiovascular Health: A Cross-Sectional Analysis Unpacking the Sequential Mediation Roles of Protein Intake and Handgrip Strength
Source: Healthcare (Basel). 2026 Jun 30;14(13):1897. doi: 10.3390/healthcare14131897 (PMC13362056; doi:10.3390/healthcare14131897)
Supplement: Supplementary file 1 [file healthcare-14-01897-s001.zip › healthcare-4313034-supplementary.pdf]

Supplementary Table S1. Sensitivity Analysis of Mediation Effects After Additional Adjustment for Smoking and Diabetes

Effect	Path	Comparison	B	Boot SE	95% CI
Total effect	X → Y	High vs Low SES	-0.949	0.281	-1.490 to -0.423
		Middle vs Low SES	-0.947	0.273	-1.490 to -0.437
Direct effect	X → Y	High vs Low SES	-0.890	0.284	-1.450 to -0.356
		Middle vs Low SES	-0.891	0.277	-1.440 to -0.376
Total indirect effect	All mediators	High vs Low SES	-0.059	0.019	-0.097 to -0.025
		Middle vs Low SES	-0.056	0.016	-0.089 to -0.029
Specific indirect effects	X → M1 → Y	High vs Low SES	-0.027	0.012	-0.056 to -0.006
		Middle vs Low SES	-0.016	0.008	-0.033 to -0.003
	X → M2 → Y	High vs Low SES	-0.029	0.015	-0.063 to -0.003
		Middle vs Low SES	-0.038	0.014	-0.067 to -0.012
	X → M1 → M2 → Y	High vs Low SES	-0.002	0.001	-0.005 to -0.001
		Middle vs Low SES	-0.001	0.001	-0.003 to -0.0003[†]

X = socioeconomic status (SES); M1 = protein intake; M2 = relative handgrip strength (HGS);

Y = estimated 10-year atherosclerotic cardiovascular disease (ASCVD) risk.

Sensitivity analyses additionally adjusted for smoking status and diabetes diagnosis.

†Values are rounded to three decimal places, except for confidence interval limits close to zero, which are shown to four decimal places where needed.
